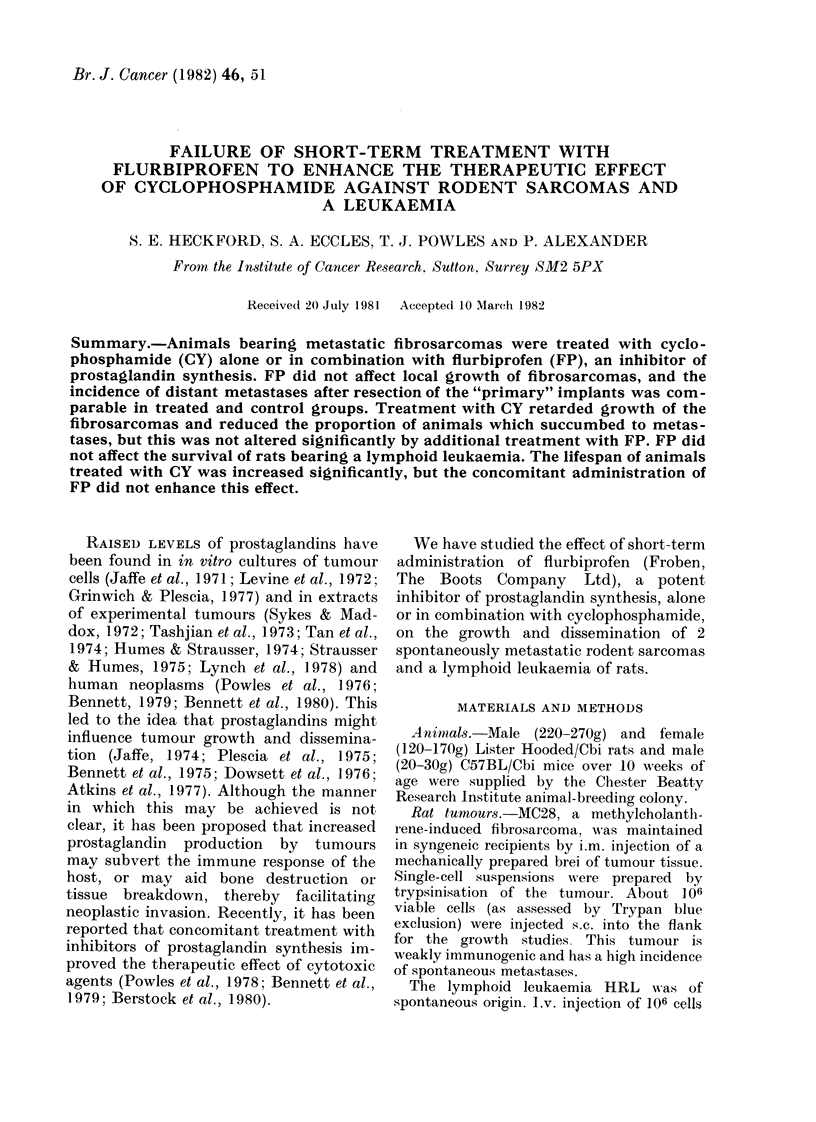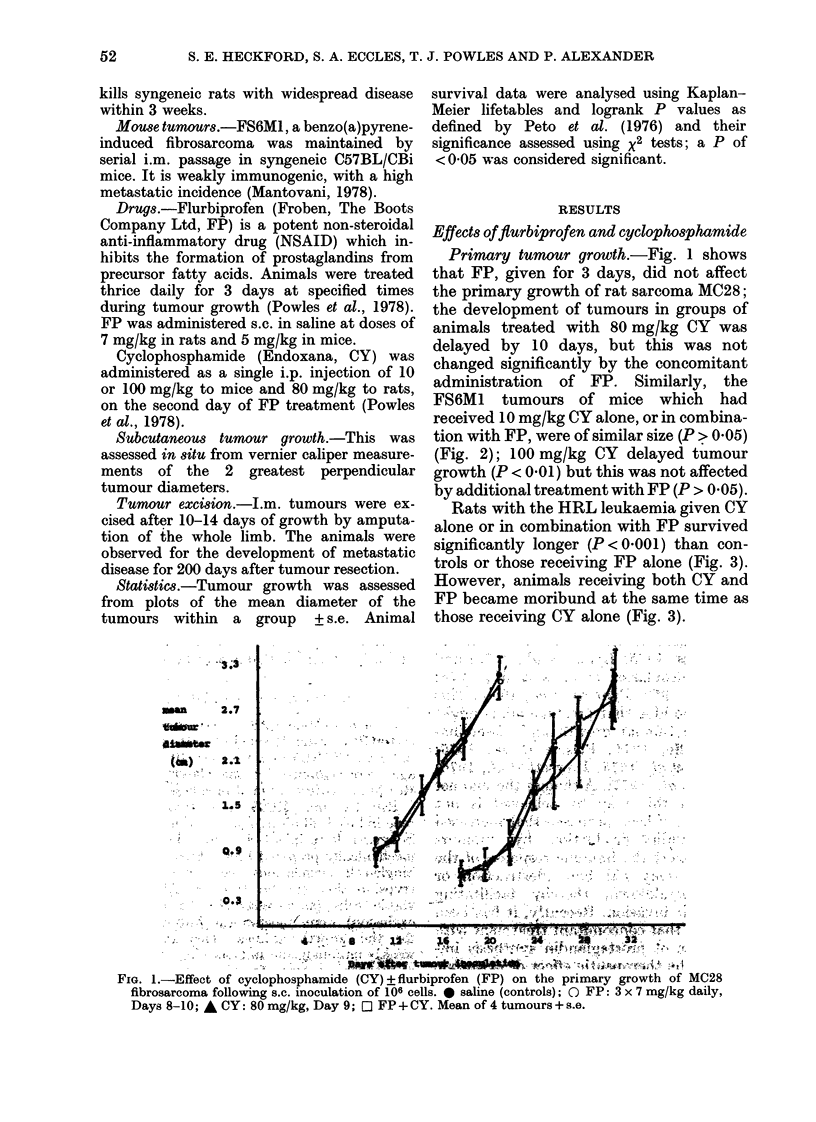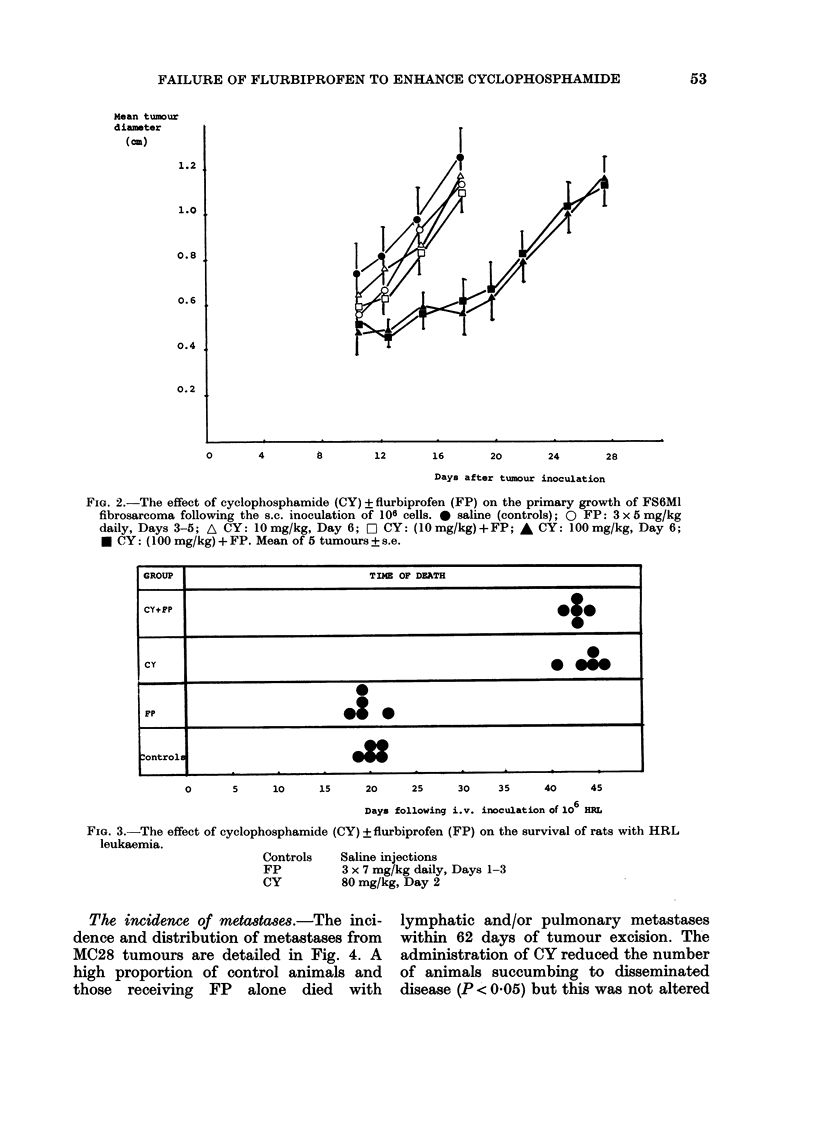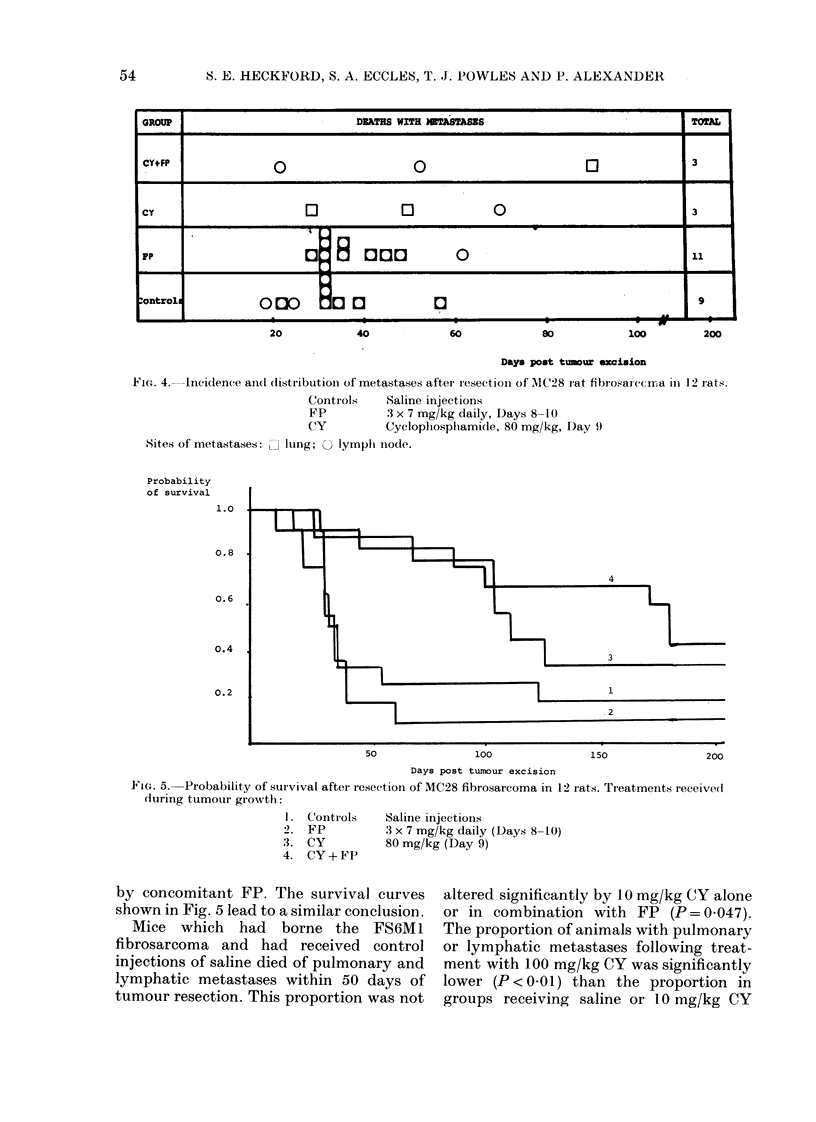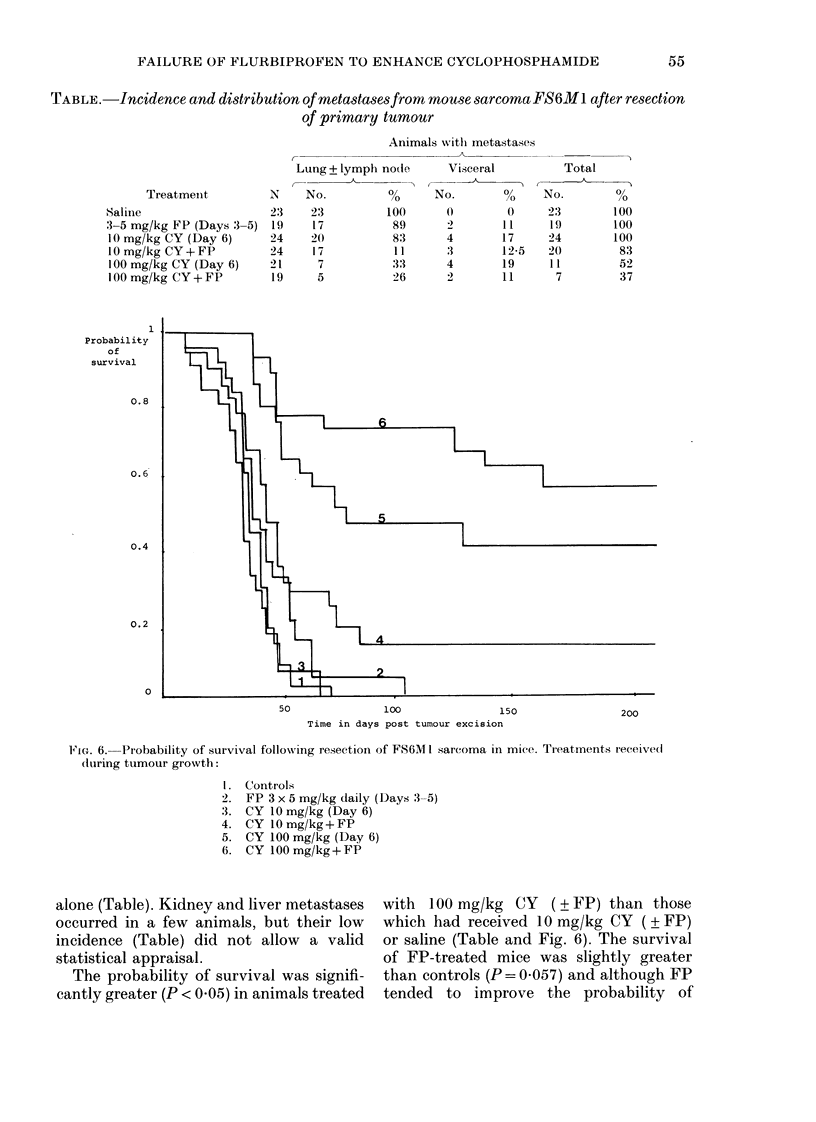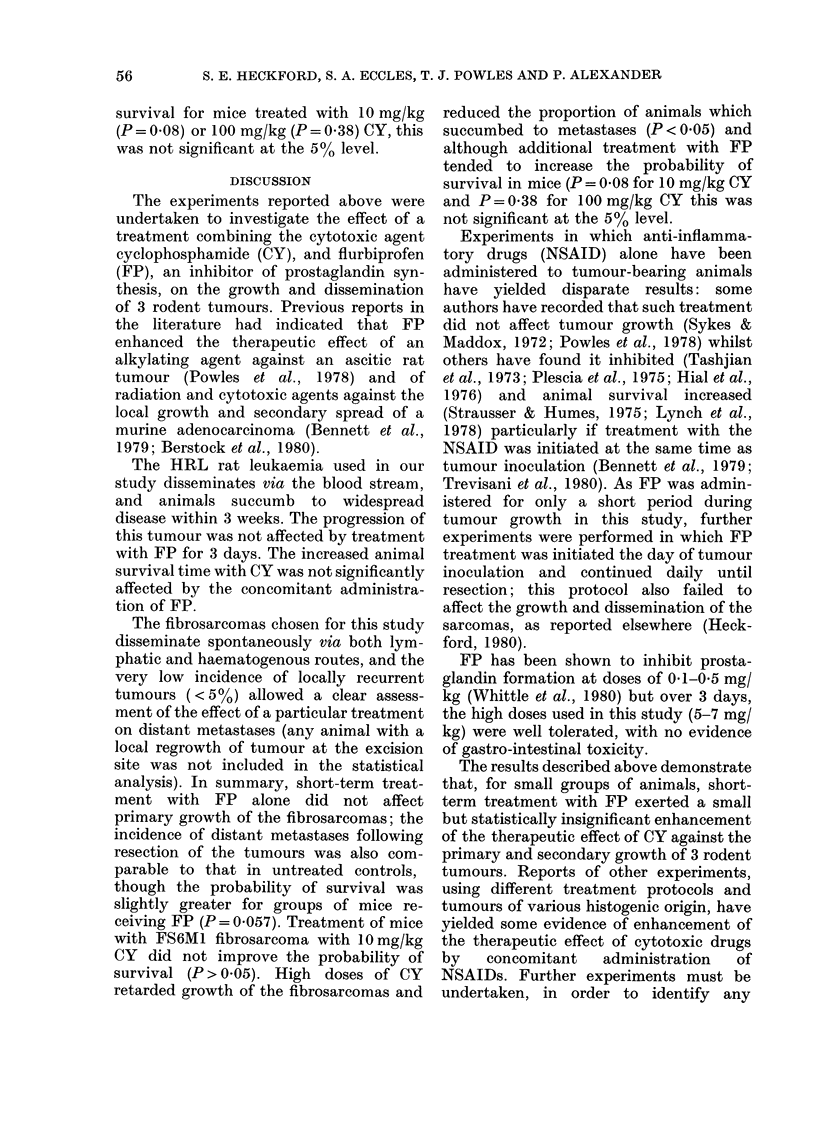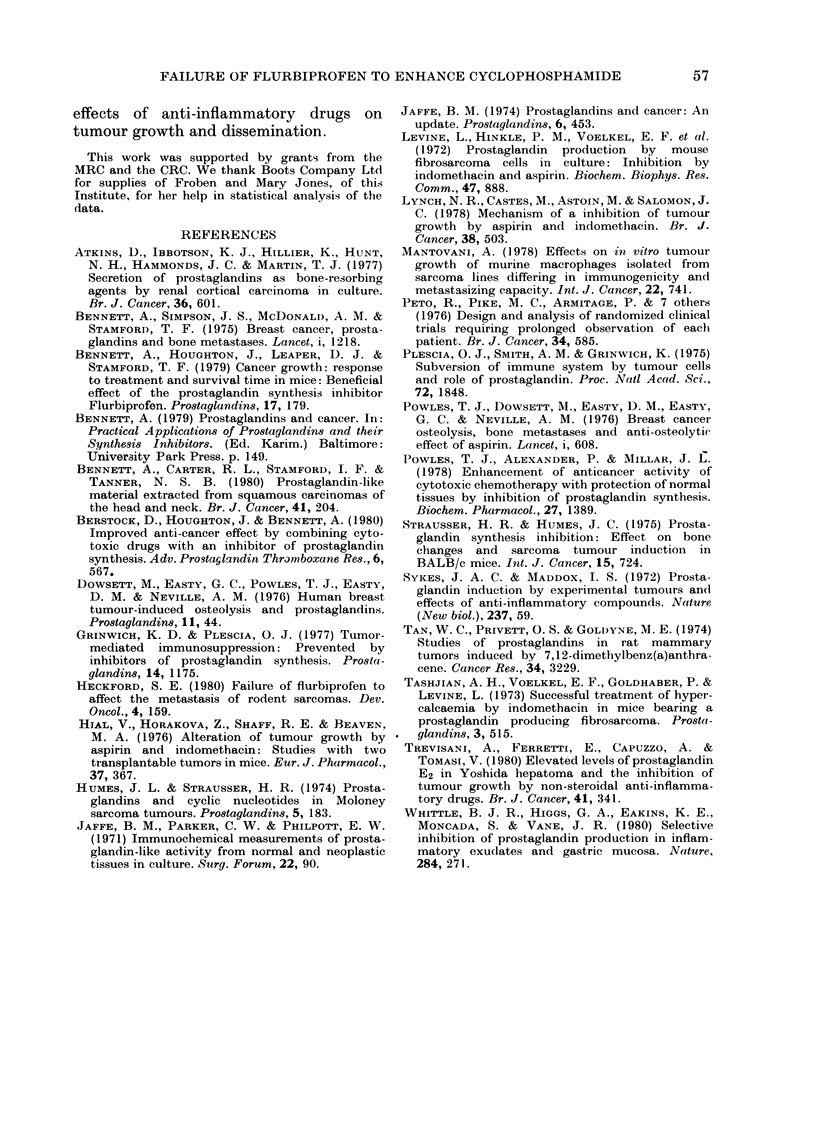# Failure of short-term treatment with flurbiprofen to enhance the therapeutic effect of cyclophosphamide against rodent sarcomas and a leukaemia.

**DOI:** 10.1038/bjc.1982.164

**Published:** 1982-07

**Authors:** S. E. Heckford, S. A. Eccles, T. J. Powles, P. Alexander

## Abstract

Animals bearing metastatic fibrosarcomas were treated with cyclophosphamide (CY) alone or in combination with flurbiprofen (FP), an inhibitor of prostaglandin synthesis. FP did not affect local growth of fibrosarcomas, and the incidence of distant metastases after resection of the "primary" implants was comparable in treated and control groups. Treatment with CY retarded growth of the fibrosarcomas and reduced the proportion of animals which succumbed to metastases, but this was not altered significantly by additional treatment with FP. FP did not affect the survival of rats bearing a lymphoid leukaemia. The lifespan of animals treated with CY was increased significantly, but the concomitant administration of FP did not enhance this effect.


*Br. J. Cancer* (1982) **46**, 51

### FAILURE OF SHORT-TERM TREATMENT WITH FLURBIPROFEN TO ENHANCE THE THERAPEUTIC EFFECT OF CYCLOPHOSPHAMIDE AGAINST RODENT SARCOMAS AND A LEUKAEMIA

S. E. HECKFORD, S. A. ECCLES, T. J. POWLES AND P. ALEXANDER

*From the Institute of Cancer Research, Sutton, Surrey SM2 5PX*

Received 20 July 1981 Accepted 10 March 1982

**Summary.—Animals bearing metastatic fibrosarcomas were treated with cyclophosphamide (CY) alone or in combination with flurbiprofen (FP), an inhibitor of prostaglandin synthesis. FP did not affect local growth of fibrosarcomas, and the incidence of distant metastases after resection of the "primary" implants was comparable in treated and control groups. Treatment with CY retarded growth of the fibrosarcomas and reduced the proportion of animals which succumbed to metastases, but this was not altered significantly by additional treatment with FP. FP did not affect the survival of rats bearing a lymphoid leukaemia. The lifespan of animals treated with CY was increased significantly, but the concomitant administration of FP did not enhance this effect.**

RAISED LEVELS of prostaglandins have been found in *in vitro* cultures of tumour cells (Jaffe *et al.*, 1971; Levine *et al.*, 1972; Grinwich & Plescia, 1977) and in extracts of experimental tumours (Sykes & Maddox, 1972; Tashjian *et al.*, 1973; Tan *et al.*, 1974; Humes & Strausser, 1974; Strausser & Humes, 1975; Lynch *et al.*, 1978) and human neoplasms (Powles *et al.*, 1976; Bennett, 1979; Bennett *et al.*, 1980). This led to the idea that prostaglandins might influence tumour growth and dissemination (Jaffe, 1974; Plescia *et al.*, 1975; Bennett *et al.*, 1975; Dowsett *et al.*, 1976; Atkins *et al.*, 1977). Although the manner in which this may be achieved is not clear, it has been proposed that increased prostaglandin production by tumours may subvert the immune response of the host, or may aid bone destruction or tissue breakdown, thereby facilitating neoplastic invasion. Recently, it has been reported that concomitant treatment with inhibitors of prostaglandin synthesis improved the therapeutic effect of cytotoxic agents (Powles *et al.*, 1978; Bennett *et al.*, 1979; Berstock *et al.*, 1980).

We have studied the effect of short-term administration of flurbiprofen (Froben, The Boots Company Ltd), a potent inhibitor of prostaglandin synthesis, alone or in combination with cyclophosphamide, on the growth and dissemination of 2 spontaneously metastatic rodent sarcomas and a lymphoid leukaemia of rats.

### MATERIALS AND METHODS

*Animals.*—Male (220–270g) and female (120–170g) Lister Hooded/Cbi rats and male (20–30g) C57BL/Cbi mice over 10 weeks of age were supplied by the Chester Beatty Research Institute animal-breeding colony.

*Rat tumours.*—MC28, a methylcholanthrene-induced fibrosarcoma, was maintained in syngeneic recipients by i.m. injection of a mechanically prepared brei of tumour tissue. Single-cell suspensions were prepared by trypsinisation of the tumour. About $10^6$ viable cells (as assessed by Trypan blue exclusion) were injected s.c. into the flank for the growth studies. This tumour is weakly immunogenic and has a high incidence of spontaneous metastases.

The lymphoid leukaemia HRL was of spontaneous origin. I.v. injection of $10^6$ cells kills syngeneic rats with widespread disease within 3 weeks.

*Mouse tumours.*—FS6M1, a benzo(a)pyrene-induced fibrosarcoma was maintained by serial i.m. passage in syngeneic C57BL/CBi mice. It is weakly immunogenic, with a high metastatic incidence (Mantovani, 1978).

*Drugs.*—Flurbiprofen (Froben, The Boots Company Ltd, FP) is a potent non-steroidal anti-inflammatory drug (NSAID) which inhibits the formation of prostaglandins from precursor fatty acids. Animals were treated thrice daily for 3 days at specified times during tumour growth (Powles *et al.*, 1978). FP was administered s.c. in saline at doses of 7 mg/kg in rats and 5 mg/kg in mice.

Cyclophosphamide (Endoxana, CY) was administered as a single i.p. injection of 10 or 100 mg/kg to mice and 80 mg/kg to rats, on the second day of FP treatment (Powles *et al.*, 1978).

*Subcutaneous tumour growth.*—This was assessed *in situ* from vernier caliper measurements of the 2 greatest perpendicular tumour diameters.

*Tumour excision.*—I.m. tumours were excised after 10–14 days of growth by amputation of the whole limb. The animals were observed for the development of metastatic disease for 200 days after tumour resection.

*Statistics.*—Tumour growth was assessed from plots of the mean diameter of the tumours within a group ± s.e. Animal survival data were analysed using Kaplan–Meier lifetables and logrank $P$ values as defined by Peto *et al.* (1976) and their significance assessed using $\chi^2$ tests; a $P$ of $<0{\cdot}05$ was considered significant.

### RESULTS

### *Effects of flurbiprofen and cyclophosphamide*

*Primary tumour growth.*—Fig. 1 shows that FP, given for 3 days, did not affect the primary growth of rat sarcoma MC28; the development of tumours in groups of animals treated with 80 mg/kg CY was delayed by 10 days, but this was not changed significantly by the concomitant administration of FP. Similarly, the FS6M1 tumours of mice which had received 10 mg/kg CY alone, or in combination with FP, were of similar size ($P > 0{\cdot}05$) (Fig. 2); 100 mg/kg CY delayed tumour growth ($P < 0{\cdot}01$) but this was not affected by additional treatment with FP ($P > 0{\cdot}05$).

Rats with the HRL leukaemia given CY alone or in combination with FP survived significantly longer ($P < 0{\cdot}001$) than controls or those receiving FP alone (Fig. 3). However, animals receiving both CY and FP became moribund at the same time as those receiving CY alone (Fig. 3).

FIG. 1.—Effect of cyclophosphamide (CY) ± flurbiprofen (FP) on the primary growth of MC28 fibrosarcoma following s.c. inoculation of $10^6$ cells. ● saline (controls); ○ FP: 3 × 7 mg/kg daily, Days 8–10; ▲ CY: 80 mg/kg, Day 9; □ FP + CY. Mean of 4 tumours + s.e.

FIG. 2.—The effect of cyclophosphamide (CY) ± flurbiprofen (FP) on the primary growth of FS6M1 fibrosarcoma following the s.c. inoculation of $10^6$ cells. ● saline (controls); ○ FP: 3 × 5 mg/kg daily, Days 3–5; △ CY: 10 mg/kg, Day 6; □ CY: (10 mg/kg) + FP; ▲ CY: 100 mg/kg, Day 6; ■ CY: (100 mg/kg) + FP. Mean of 5 tumours ± s.e.

FIG. 3.—The effect of cyclophosphamide (CY) ± flurbiprofen (FP) on the survival of rats with HRL leukaemia.

| | |
|---|---|
| Controls | Saline injections |
| FP | 3 × 7 mg/kg daily, Days 1–3 |
| CY | 80 mg/kg, Day 2 |

*The incidence of metastases.*—The incidence and distribution of metastases from MC28 tumours are detailed in Fig. 4. A high proportion of control animals and those receiving FP alone died with lymphatic and/or pulmonary metastases within 62 days of tumour excision. The administration of CY reduced the number of animals succumbing to disseminated disease ($P < 0{\cdot}05$) but this was not altered

FIG. 4.—Incidence and distribution of metastases after resection of MC28 rat fibrosarcoma in 12 rats.

| | |
|---|---|
| Controls | Saline injections |
| FP | 3 x 7 mg/kg daily, Days 8–10 |
| CY | Cyclophosphamide, 80 mg/kg, Day 9 |

Sites of metastases: □ lung; ○ lymph node.

FIG. 5.—Probability of survival after resection of MC28 fibrosarcoma in 12 rats. Treatments received during tumour growth:

| | | |
|---|---|---|
| 1. | Controls | Saline injections |
| 2. | FP | 3 x 7 mg/kg daily (Days 8–10) |
| 3. | CY | 80 mg/kg (Day 9) |
| 4. | CY + FP | |

by concomitant FP. The survival curves shown in Fig. 5 lead to a similar conclusion.

Mice which had borne the FS6M1 fibrosarcoma and had received control injections of saline died of pulmonary and lymphatic metastases within 50 days of tumour resection. This proportion was not altered significantly by 10 mg/kg CY alone or in combination with FP ($P = 0{\cdot}047$). The proportion of animals with pulmonary or lymphatic metastases following treatment with 100 mg/kg CY was significantly lower ($P < 0{\cdot}01$) than the proportion in groups receiving saline or 10 mg/kg CY

TABLE.—*Incidence and distribution of metastases from mouse sarcoma FS6M1 after resection of primary tumour*

| | | Animals with metastases | | | | | |
|---|---|---|---|---|---|---|---|
| | | Lung ± lymph node | | Visceral | | Total | |
| Treatment | N | No. | % | No. | % | No. | % |
| Saline | 23 | 23 | 100 | 0 | 0 | 23 | 100 |
| 3–5 mg/kg FP (Days 3–5) | 19 | 17 | 89 | 2 | 11 | 19 | 100 |
| 10 mg/kg CY (Day 6) | 24 | 20 | 83 | 4 | 17 | 24 | 100 |
| 10 mg/kg CY + FP | 24 | 17 | 11 | 3 | 12·5 | 20 | 83 |
| 100 mg/kg CY (Day 6) | 21 | 7 | 33 | 4 | 19 | 11 | 52 |
| 100 mg/kg CY + FP | 19 | 5 | 26 | 2 | 11 | 7 | 37 |

FIG. 6.—Probability of survival following resection of FS6M1 sarcoma in mice. Treatments received during tumour growth:

1. Controls
2. FP 3 × 5 mg/kg daily (Days 3–5)
3. CY 10 mg/kg (Day 6)
4. CY 10 mg/kg + FP
5. CY 100 mg/kg (Day 6)
6. CY 100 mg/kg + FP

alone (Table). Kidney and liver metastases occurred in a few animals, but their low incidence (Table) did not allow a valid statistical appraisal.

The probability of survival was significantly greater ($P < 0{\cdot}05$) in animals treated with 100 mg/kg CY (± FP) than those which had received 10 mg/kg CY (± FP) or saline (Table and Fig. 6). The survival of FP-treated mice was slightly greater than controls ($P = 0{\cdot}057$) and although FP tended to improve the probability of survival for mice treated with 10 mg/kg ($P = 0{\cdot}08$) or 100 mg/kg ($P = 0{\cdot}38$) CY, this was not significant at the 5% level.

### DISCUSSION

The experiments reported above were undertaken to investigate the effect of a treatment combining the cytotoxic agent cyclophosphamide (CY), and flurbiprofen (FP), an inhibitor of prostaglandin synthesis, on the growth and dissemination of 3 rodent tumours. Previous reports in the literature had indicated that FP enhanced the therapeutic effect of an alkylating agent against an ascitic rat tumour (Powles *et al.*, 1978) and of radiation and cytotoxic agents against the local growth and secondary spread of a murine adenocarcinoma (Bennett *et al.*, 1979; Berstock *et al.*, 1980).

The HRL rat leukaemia used in our study disseminates *via* the blood stream, and animals succumb to widespread disease within 3 weeks. The progression of this tumour was not affected by treatment with FP for 3 days. The increased animal survival time with CY was not significantly affected by the concomitant administration of FP.

The fibrosarcomas chosen for this study disseminate spontaneously *via* both lymphatic and haematogenous routes, and the very low incidence of locally recurrent tumours (< 5%) allowed a clear assessment of the effect of a particular treatment on distant metastases (any animal with a local regrowth of tumour at the excision site was not included in the statistical analysis). In summary, short-term treatment with FP alone did not affect primary growth of the fibrosarcomas; the incidence of distant metastases following resection of the tumours was also comparable to that in untreated controls, though the probability of survival was slightly greater for groups of mice receiving FP ($P = 0{\cdot}057$). Treatment of mice with FS6M1 fibrosarcoma with 10 mg/kg CY did not improve the probability of survival ($P > 0{\cdot}05$). High doses of CY retarded growth of the fibrosarcomas and reduced the proportion of animals which succumbed to metastases ($P < 0{\cdot}05$) and although additional treatment with FP tended to increase the probability of survival in mice ($P = 0{\cdot}08$ for 10 mg/kg CY and $P = 0{\cdot}38$ for 100 mg/kg CY this was not significant at the 5% level.

Experiments in which anti-inflammatory drugs (NSAID) alone have been administered to tumour-bearing animals have yielded disparate results: some authors have recorded that such treatment did not affect tumour growth (Sykes & Maddox, 1972; Powles *et al.*, 1978) whilst others have found it inhibited (Tashjian *et al.*, 1973; Plescia *et al.*, 1975; Hial *et al.*, 1976) and animal survival increased (Strausser & Humes, 1975; Lynch *et al.*, 1978) particularly if treatment with the NSAID was initiated at the same time as tumour inoculation (Bennett *et al.*, 1979; Trevisani *et al.*, 1980). As FP was administered for only a short period during tumour growth in this study, further experiments were performed in which FP treatment was initiated the day of tumour inoculation and continued daily until resection; this protocol also failed to affect the growth and dissemination of the sarcomas, as reported elsewhere (Heckford, 1980).

FP has been shown to inhibit prostaglandin formation at doses of 0·1–0·5 mg/kg (Whittle *et al.*, 1980) but over 3 days, the high doses used in this study (5–7 mg/kg) were well tolerated, with no evidence of gastro-intestinal toxicity.

The results described above demonstrate that, for small groups of animals, short-term treatment with FP exerted a small but statistically insignificant enhancement of the therapeutic effect of CY against the primary and secondary growth of 3 rodent tumours. Reports of other experiments, using different treatment protocols and tumours of various histogenic origin, have yielded some evidence of enhancement of the therapeutic effect of cytotoxic drugs by concomitant administration of NSAIDs. Further experiments must be undertaken, in order to identify any effects of anti-inflammatory drugs on tumour growth and dissemination.

This work was supported by grants from the MRC and the CRC. We thank Boots Company Ltd for supplies of Froben and Mary Jones, of this Institute, for her help in statistical analysis of the data.

### REFERENCES

ATKINS, D., IBBOTSON, K. J., HILLIER, K., HUNT, N. H., HAMMONDS, J. C. & MARTIN, T. J. (1977) Secretion of prostaglandins as bone-resorbing agents by renal cortical carcinoma in culture. *Br. J. Cancer*, **36**, 601.

BENNETT, A., SIMPSON, J. S., MCDONALD, A. M. & STAMFORD, T. F. (1975) Breast cancer, prostaglandins and bone metastases. *Lancet*, i, 1218.

BENNETT, A., HOUGHTON, J., LEAPER, D. J. & STAMFORD, T. F. (1979) Cancer growth: response to treatment and survival time in mice: Beneficial effect of the prostaglandin synthesis inhibitor Flurbiprofen. *Prostaglandins*, **17**, 179.

BENNETT, A. (1979) Prostaglandins and cancer. In: *Practical Applications of Prostaglandins and their Synthesis Inhibitors*. (Ed. Karim.) Baltimore: University Park Press. p. 149.

BENNETT, A., CARTER, R. L., STAMFORD, I. F. & TANNER, N. S. B. (1980) Prostaglandin-like material extracted from squamous carcinomas of the head and neck. *Br. J. Cancer*, **41**, 204.

BERSTOCK, D., HOUGHTON, J. & BENNETT, A. (1980) Improved anti-cancer effect by combining cytotoxic drugs with an inhibitor of prostaglandin synthesis. *Adv. Prostaglandin Thromboxane Res.*, **6**, 567.

DOWSETT, M., EASTY, G. C., POWLES, T. J., EASTY, D. M. & NEVILLE, A. M. (1976) Human breast tumour-induced osteolysis and prostaglandins. *Prostaglandins*, **11**, 44.

GRINWICH, K. D. & PLESCIA, O. J. (1977) Tumor-mediated immunosuppression: Prevented by inhibitors of prostaglandin synthesis. *Prostaglandins*, **14**, 1175.

HECKFORD, S. E. (1980) Failure of flurbiprofen to affect the metastasis of rodent sarcomas. *Dev. Oncol.*, **4**, 159.

HIAL, V., HORAKOVA, Z., SHAFF, R. E. & BEAVEN, M. A. (1976) Alteration of tumour growth by aspirin and indomethacin: Studies with two transplantable tumors in mice. *Eur. J. Pharmacol.*, **37**, 367.

HUMES, J. L. & STRAUSSER, H. R. (1974) Prostaglandins and cyclic nucleotides in Moloney sarcoma tumours. *Prostaglandins*, **5**, 183.

JAFFE, B. M., PARKER, C. W. & PHILPOTT, E. W. (1971) Immunochemical measurements of prostaglandin-like activity from normal and neoplastic tissues in culture. *Surg. Forum*, **22**, 90.

JAFFE, B. M. (1974) Prostaglandins and cancer: An update. *Prostaglandins*, **6**, 453.

LEVINE, L., HINKLE, P. M., VOELKEL, E. F. *et al.* (1972) Prostaglandin production by mouse fibrosarcoma cells in culture: Inhibition by indomethacin and aspirin. *Biochem. Biophys. Res. Comm.*, **47**, 888.

LYNCH, N. R., CASTES, M., ASTOIN, M. & SALOMON, J. C. (1978) Mechanism of a inhibition of tumour growth by aspirin and indomethacin. *Br. J. Cancer*, **38**, 503.

MANTOVANI, A. (1978) Effects on *in vitro* tumour growth of murine macrophages isolated from sarcoma lines differing in immunogenicity and metastasizing capacity. *Int. J. Cancer*, **22**, 741.

PETO, R., PIKE, M. C., ARMITAGE, P. & 7 others (1976) Design and analysis of randomized clinical trials requiring prolonged observation of each patient. *Br. J. Cancer*, **34**, 585.

PLESCIA, O. J., SMITH, A. M. & GRINWICH, K. (1975) Subversion of immune system by tumour cells and role of prostaglandin. *Proc. Natl Acad. Sci.*, **72**, 1848.

POWLES, T. J., DOWSETT, M., EASTY, D. M., EASTY, G. C. & NEVILLE, A. M. (1976) Breast cancer osteolysis, bone metastases and anti-osteolytic effect of aspirin. *Lancet*, i, 608.

POWLES, T. J., ALEXANDER, P. & MILLAR, J. L. (1978) Enhancement of anticancer activity of cytotoxic chemotherapy with protection of normal tissues by inhibition of prostaglandin synthesis. *Biochem. Pharmacol.*, **27**, 1389.

STRAUSSER, H. R. & HUMES, J. C. (1975) Prostaglandin synthesis inhibition: Effect on bone changes and sarcoma tumour induction in BALB/c mice. *Int. J. Cancer*, **15**, 724.

SYKES, J. A. C. & MADDOX, I. S. (1972) Prostaglandin induction by experimental tumours and effects of anti-inflammatory compounds. *Nature* (*New biol.*), **237**, 59.

TAN, W. C., PRIVETT, O. S. & GOLDYNE, M. E. (1974) Studies of prostaglandins in rat mammary tumors induced by 7,12-dimethylbenz(a)anthracene. *Cancer Res.*, **34**, 3229.

TASHJIAN, A. H., VOELKEL, E. F., GOLDHABER, P. & LEVINE, L. (1973) Successful treatment of hypercalcaemia by indomethacin in mice bearing a prostaglandin producing fibrosarcoma. *Prostaglandins*, **3**, 515.

TREVISANI, A., FERRETTI, E., CAPUZZO, A. & TOMASI, V. (1980) Elevated levels of prostaglandin $E_2$ in Yoshida hepatoma and the inhibition of tumour growth by non-steroidal anti-inflammatory drugs. *Br. J. Cancer*, **41**, 341.

WHITTLE, B. J. R., HIGGS, G. A., EAKINS, K. E., MONCADA, S. & VANE, J. R. (1980) Selective inhibition of prostaglandin production in inflammatory exudates and gastric mucosa. *Nature*, **284**, 271.